# Association Between Serum Phosphate Levels and Progression of Aortic Stenosis

**DOI:** 10.1016/j.jacadv.2024.101096

**Published:** 2024-07-06

**Authors:** Alexandre Candellier, Momar Diouf, Yohann Bohbot, Youssef Bennis, Agnes Pasquet, Eric Goffin, Lucie Hénaut, Gabriel Choukroun, Jean-Louis Vanoverschelde, Christophe Tribouilloy

**Affiliations:** a Amiens University Hospital, Amiens, France; b Jules Verne University of Picardie, Amiens, France; c Cliniques Universitaires Saint-Luc, Brussels, Belgium

Aortic stenosis (AS) is a complex disease process involving fibrosis and calcification of the aortic valve leaflets. As there is no known effective medical therapy, the identification of modifiable risk factors for progression is a key objective. Epidemiological and pathophysiological evidence suggests a role of phosphate in the pathogenesis of cardiac valve calcification.^1^, ^2^, ^3^ We aimed to investigate the relationship between serum phosphate levels and the progression of AS.

All consecutive patients diagnosed with AS (Vmax ≥2.5 m/s) and preserved left ventricular ejection fraction in two tertiary centers (Amiens, Brussels) were included in an electronic database. The methodology has been described elsewhere.^4^ Briefly, we retrospectively identified patients with ≥2 transthoracic echocardiograms (TTEs) at least 1 year apart and an available fasting serum measurement of phosphate, serum nonionized total calcium levels, and creatinine value at enrollment. Patients under dialysis, kidney graft recipients, patients with a history of hypoparathyroidism, and those receiving phosphate-lowering drugs were excluded. Groups were stratified according to serum phosphate levels ≤3.0 mg/dL (n = 149), 3.01 to 3.50 mg/dl (n = 177), 3.51 to 4.0 mg/dL (n = 123), and >4.0 mg/dl (n = 40), as previously described.^1^ The primary outcome was AS progression in each phosphate-level group evaluated using a multivariable linear mixed effect model based on repeated measurements of aortic valve area (AVA) from subsequent TTEs of the same subject. Annualized progression rate was obtained by introducing the interaction between the variable that defines the phosphate groups and time. We entered covariates considered to have a potential prognostic impact into the model on an epidemiological basis. They were age, sex, body surface area, history of hypertension, dyslipidemia, diabetes, smoking, coronary artery disease, atrial fibrillation, AVA at inclusion, and estimated glomerular filtration rate determined using the 2021 CKD-EPI (Chronic Kidney Disease Epidemiology Collaboration) equation. The association between serum phosphate level and AS progression was assessed by linear regression. Restricted cubic spline logistic models were used to assess the adjusted association between serum phosphate levels (as a continuous variable) and the rapid progression of AS (defined as AVA reduction >0.07 cm^2^/year [median of the cohort]). Each participating center received Institutional Review Board approval (DR-2015-349). The study was conducted in accordance with the revised Declaration of Helsinki.

Our study population consisted of 489 patients (46.6% women, mean age 75.2 ± 10.4 years). The median serum phosphate level was 3.28 (IQR: 2.97-3.62) mg/dL. There were no differences between the serum phosphate groups in terms of baseline characteristics, comorbidities, or aortic valve parameters (all interaction *P* values ≥0.17), except for sex (*P* < 0.01).

**Figure 1:**
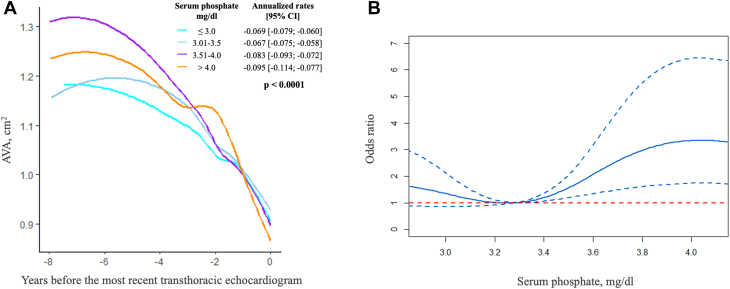
Progression of Aortic Stenosis According to Serum Phosphate Levels (A) Annualized progression rate of AVA expressed as mean and 95% CI and according to serum phosphate group by the adjusted linear mixed effects model. (B) Restricted cubic spline curve showing adjusted odds ratios (solid line) and 95% CI (dashed blue lines) for rapid progression of AS with serum phosphate level. AS = aortic stenosis; AVA = aortic valve area.

The median follow-up between the first and last echocardiograms was 35 (IQR: 22-56) months and 318 patients (65.0%) had ≥3 TTEs. The overall mean annualized progression rate of AVA was −0.078 (95% CI: −0.083 to −0.073) cm^2^/year. Patients in the serum phosphate groups ≤3.0 mg/dl and 3.01 to 3.50 mg/dL had significantly slower mean AVA narrowing (−0.069 [95% CI: −0.079 to −0.060] and −0.067 [95% CI: −0.075 to −0.058] cm^2^/year, respectively) than patients in the 3.51 to 4.0 mg/dL phosphate group (−0.083 [95% CI: −0.093 to −0.072] cm^2^/year) or >4.0 mg/dL phosphate group (−0.095 [95% CI: −0.114 to −0.077] cm^2^/year) (*P* < 0.001) in the adjusted linear mixed effect model (Figure 1A). In multivariable linear regression analysis, a higher serum phosphate level was independently associated with annualized AVA narrowing (standardized β = −0.11, *P* = 0.02) and remained significantly associated with the values for percent reduction in AVA per year (*P* = 0.004). Higher serum phosphate level was also independently associated with annualized increased in Vmax (standardized β = 0.16, *P* = 0.001) and mean gradient (standardized β = 0.15, *P* = 0.001).

Fully adjusted restricted cubic spline curves showed no significant association between rapid progression of AS and phosphate levels under the median value of the cohort. Conversely, the odds ratio for rapid progression markedly increased for serum phosphate values > 3.4 mg/dL (Figure 1B).

In multivariable regression analysis, the initial serum calcium-phosphate product was significantly related to absolute (*P* = 0.04) and relative (*P* = 0.01) reduction in AVA per year. However, serum calcium levels (corrected or uncorrected for serum albumin) were not associated with AS progression (all *P* ≥ 0.51).

We described in this pilot study a significant association between serum phosphate levels and AS progression, independent of kidney function in a population-based cohort. A key role of phosphate, independent of estimated glomerular filtration rate or calcium levels, has already been suggested in the development of AS.^1^^,^^2^ In vitro, higher phosphate levels within the normal range can trigger osteogenic transformation and mineralization of cultured valvular interstitial cells.^3^ Our findings support the potential of targeting phosphate to slow down AS progression. Sevelamer, a phosphate binder, may be a promising interventional agent to safely lower elevated phosphate levels toward the normal range in AS patients.^5^ Due to the retrospective nature of our work, the observed association might have been confounded by unmeasurable factors. Unfortunately, serum levels of parathormone and 25-(OH)-vitamin-D which may play biological roles in phosphate metabolism were not evaluated in our study.

In conclusion, phosphate may be a novel independent modifiable risk factor for the progression of AS.
